# Mechanical Properties and Shrinkage Behavior of Concrete-Containing Graphene-Oxide Nanosheets

**DOI:** 10.3390/ma13030590

**Published:** 2020-01-27

**Authors:** Zengshun Chen, Yemeng Xu, Jianmin Hua, Xu Wang, Lepeng Huang, Xiao Zhou

**Affiliations:** 1 School of Civil Engineering, Chongqing University, Chongqing 400033, China; zchenba@connect.ust.hk (Z.C.); xu.ym@cqu.edu.cn (Y.X.);; 2 Department of Civil and Environmental Engineering, The Hong Kong University of Science and Technology, Hong Kong, China; 3 School of Civil Engineering, Chongqing Jiaotong University, Chongqing 400074, China; xuwang@cqjtu.edu.cn (X.W.); xiaozhou@cqjtu.edu.cn (X.Z.)

**Keywords:** concrete, graphene oxide, mechanical behavior, shrinkage

## Abstract

Graphene oxide (GO) has been widely used as an additive due to its numerous unique properties. In this study, the compressive strength, flexural strength and elasticity modulus of concrete containing 0.02 wt%, 0.05 wt % and 0.08 wt % GO, and its dry shrinkage performance have been experimentally investigated. After the sample preparation, apparatus for compression test and flexural test were used to test the relevant properties of concrete containing GO. The dial indicators were used to measure the shrinkage of samples. The results indicate that GO can considerably improve the compressive strength, flexural strength, and elasticity modulus of concrete at the concrete age of 28 days by 4.04–12.65%, 3.8–7.38%, and 3.92–10.97%, respectively, which are substantially smaller than the increment at the age of 3 d by 5.02–21.51%, 4.25–13.06%, and 6.07–27.45% under a water-cement ratio of 0.35. It was also found that GO can increase the shrinkage strain of concrete. For example, at the age of 60 days, 0.02 wt%, 0.05 wt% and 0.08 wt% GO can increase the shrinkage strain of ordinary concrete by 1.99%, 5.79% and 7.45% respectively under a water-cement ratio of 0.49. The study has advanced our understanding on mechanical and shrinkage behavior of concrete containing GO.

## 1. Introduction

Since the emerging of concrete, researchers have been devoted to obtaining concrete with higher strength and better performance [1,2,3]. Graphene oxide (GO) and reduced-graphene oxide (rGO) were found useful to enhance the thermal conductivity, Young’s modulus and mechanical properties of composites [4,5,6]. Rafiee et al. [4] investigated the thermal properties of doubly reinforced fiberglass/epoxy composites with graphene oxide and reduced graphene oxide. The results showed that the introduction of GO and rGO can enhance the thermal conductivity. Zhu et al. [5] investigated the mechanical properties of monolayer graphene and few-layer graphene. It was found that defect-free graphene has a Young’s modulus of 1.0 TPa and a fracture strength of 130 GPa, which is much higher than those of ordinary concrete. Rafiee et al. [6] employed GO and its thermally reduced version (rGO) to modify the epoxy matrix and the surface of glass fibers and results indicated that the introduction of GO and rGO enhanced the mechanical properties.

In addition, it is discovered that graphene oxide (GO) and its derivatives, as an additive, can be used for repairing cracks and pores in concrete [7,8]. Compared with reduced graphene oxide and graphene, GO has more hydrophilic, which has made it widely used [9,10]. Besides, it is found that GO can not only universally improve the workability, cement hydration, micro-development, mechanical strength and material transfer features of cement-based composite materials [10,11], but also enhance the mechanical properties [12,13] and shrinkage behavior [9,14] of cement-based materials.

Many studies have focused on the effect of GO on mechanical properties of concrete [9]. Wang et al. [15] have found that the bending strength of cement paste could be significantly improved when incorporated with 0.03 wt% GO. Li et al. [16] discovered that the addition of 0.04 wt% GO could increase the compressive strength of silicate cement paste the most. Li et al. [17] argued that the GO agglomerate could enhance the tensile property of cement-based materials. However, there are no consistent opinions on the enhancement effects of GO. Li et al. [18] found that GO at dosages of 0.02 wt% and 0.04 wt% did not show a huge difference in the influence on cement-based nanocomposite materials, which was possibly because the strong Van der Waals’ force under nanoscale. They found that the influence of GO on compressive strength was greater than that on tensile strength. Qin et al. [19] have found that when 0.01 wt% and 0.03 wt% GO was added into concrete containing a certain amount of fly ash, the influence on flexural strength was greater than the influence on compressive strength. Chen et al. [20] discovered similar trends for GO carbon-fiber cement composites. Li et al. [21] have measured the flexural strength, compressive strength and tensile strength of cement paste with GO at dosage of 0.02 wt% and 0.04 wt% at the age of 3 d, 7 d and 28 d. According to the results, GO nanosheets had the most obvious influence on compressive strength, followed by the influence on flexural strength and tensile strength. When it comes to the dosage of GO, many researchers have found that adding slight GO (e.g. 0.02 wt% and 0.08 wt%) has a significant effect on mechanical properties of cement paste, but when the added GO nanosheet level is higher than 0.04 wt%, it would reduce the bending strength of cement paste [7,14,16].

From a three-dimensional perspective, Long et al. [22] have demonstrated that GO had a certain enhancement effect on the elasticity modulus of cement paste. He et al. [23] found that the water suspension of GO nanosheets could help maintaining the moisture in cement paste and reducing the shrinkage of concrete. Mokhtar et al. [24] pointed out that the pores in concrete had a huge influence on its strength, durability and shrinkage. It was argued that the micro pores (<50 nm) might influence the dry shrinkage and creep. Pei et al. [14] found that 0.06% GO (by weight of cement) could significantly reduce the shrinkage strain of magnesium-potassium-phosphate cement, and make it achieve the optimal workability. 

Kang et al. [25] explored the influence of GO on tricalcium silicate (C_3_S). It was widely believed that GO can promote the hydration reaction of cement-based materials [25,26,27,28]. The pore structure and microstructure of cement-based materials doped with graphene oxide were also studied [17,29]. It was believed that GO can refine pore structure. Long et al. [30] discovered that GO could restrain the formation of pores within the scope of 1–5 nm. Theoretically speaking, from a structural point of view, the nanosheet structure may influence the mechanical properties of nanocomposites. Vijayaraghavan et al. [31] have performed a comprehensive investigation for tensile loading characteristics of hybrid boron nitride–carbon (BN-C) reinforced PE nanocomposites. It was found the tensile load-carrying capacity of the BN-C nanocomposites was considerably influenced by the lattice structure and geometry of the reinforcing BN-C nanosheet.

Although above studies have investigated mechanical and physical properties of cement-based materials with GO, there is no consistent conclusion for mechanical properties and shrinkage effect of GO concrete. Specially, the influence level of GO on compressive strength and flexural strength remains controversial. Therefore, in addition to the exploration about drying shrinkage effect of GO concrete, this study also aims to study compressive strength and flexural strength of concrete containing GO nanosheets.

## 2. Experimental Setups

### 2.1. Mechanical Properties Test for Concrete Containing GO Nanosheets

#### 2.1.1. Material and Instrument

**Figure 1:**
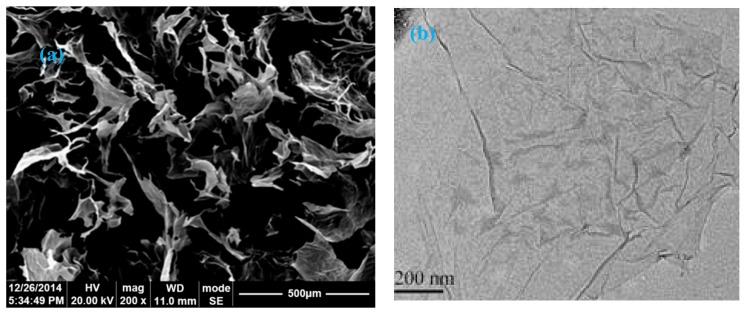
SEM and TEM image [32]: (**a**) SEM image of multilayer GO material; (**b**)TEM image of GO nanosheet.

The cement used to make concrete was ordinary Portland cement P.O 42.5. Details of the material components and parameters can be found in [32]. A scanning electron microscope (SEM) image and a transmission electron microscope (TEM) image of a typical GO nanosheet [32] are shown in Figure 1a,b, respectively.

**Figure 2:**
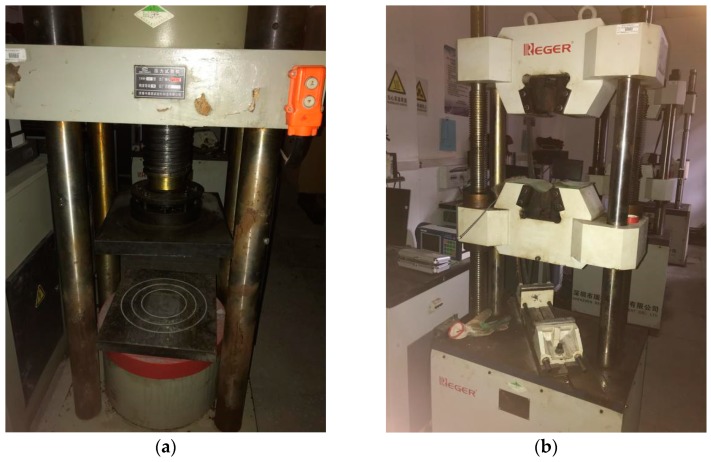
Laboratory apparatus: (**a**) apparatus for compression test; (**b**) apparatus for flexural test.

The YAW-2000 compression-testing machine provided by Center of Engineering Materials at Chongqing University was used as the concrete compression strength instrument, as shown in Figure 2a. Its technical parameters are presented in Table 1. The RE-8060G electro-hydraulic servo universal testing machine of microprocessor control provided by Center of Engineering Materials at Chongqing University was used for the experimental apparatus for concrete flexural strength, as shown in Figure 2b. Its technical parameters are presented in Table 2.

#### 2.1.2. Mixture Ratio

In the experiment, four groups of C50 concrete samples were prepared, and they were numbered as PC50, GOC50-1, GOC50-2 and GOC50-3, where PC50 was the ordinary concrete and GOC50-1, GOC50-2, GOC50-3 were concrete added with 0.02 wt%, 0.05 wt%, 0.08 wt% GO, respectively. 36 samples were included in each group. The water-cement ratio (w/c) was 0.35. The specific mixture ratio is shown in Table 3.

#### 2.1.3. Sample Preparation and Test

The preparation method for samples is stated in the *Standard for Test Method of Mechanical Properties of on Ordinary Concrete* (GB/T 50081-2002). The dimension of the samples for compressive strength tests was 100 mm × 100 mm × 100 mm, and the dimension of the samples for flexural strength tests was 100 mm × 100 mm × 400 mm, and the dimension of the samples for elasticity modulus tests under static pressure was 150 mm × 150 mm × 300 mm.

Before preparation for the samples, the GO powder was dissolved in water, and a polycarboxylate superplasticizer was added into the water solution of GO to make the GO powder dissolve in water better. Subsequently, the solution was mixed with other binding materials and aggregates. The concrete mixture was put into the test mold and the slice was used to smash the internal wall of the test mold. The mixture should be higher than the test mold orifice. After loading, the test mold was placed on the shaking table for vibration until paste appeared on the surface. When the samples were shaped up, a water-proof thin film was attached to the surface to prevent water desorption. During the preparation of the mixture, abnormal phenomenon didn’t been observed. The samples were numbered and the mold was removed after 24 h. Then, the samples were immediately moved into a standard curing room with the temperature of 20 ± 2 ℃ and relative humidity of above 95%.

During the compressive strength test, the sample was taken out from the curing room, and the surface was dried. Then the sample was placed in the center of lower platen for loading with the loading speed of 0.07 MPa/s according to the *Standard for Test Method of Mechanical Properties of on Ordinary Concrete* (GB/T 50081-2002). When the sample began to deform rapidly and approached destruction, adjustment of the tester accelerator was stopped until the sample was destroyed. Then the data were recorded.

**Figure 3:**
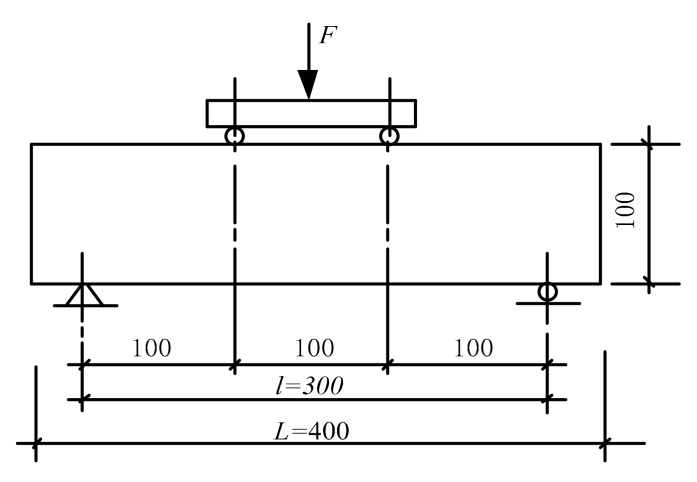
Flexural test diagram.

During the flexural test, the sample was taken out, and the surface was dried. Then the sample was installed according to Figure 3. The stand leg was a fixed hinge and the others were rolling pivots just like the standard recommended. The sample side was used as the pressure-bearing surface to make the contact surface of cylinder with support and pressure-bearing surface stable. The load was applied continuously and uniformly to the sample with a loading speed of 0.07 MPa/s according to the Standard for Test Method of Mechanical Properties of on Ordinary Concrete (GB/T 50081-2002) until the sample was destroyed. The failure load data and the fracture location at the lower edge of the sample were recorded.

Before the static pressure test, the sample was taken out from the curing room, and the surface of the sample and the upper and lower bearing plates of the apparatus were wiped up. Static pressure test was conducted for 3 samples and axial compression test was conducted for 3 samples at the same time. The data of axial compression test were used to verify the validity of the data of static pressure test. Then the apparatus was started to load till the benchmark stress was 0.5 MPa. The loading duration was kept for 60 s, and the deformation at various measurement points was recorded. Then the load was increased to 1/3 of axial compressive strength uniformly. The loading duration was kept for 60 s, and the deformation at various measurement points was recorded. 

**Figure 4:**
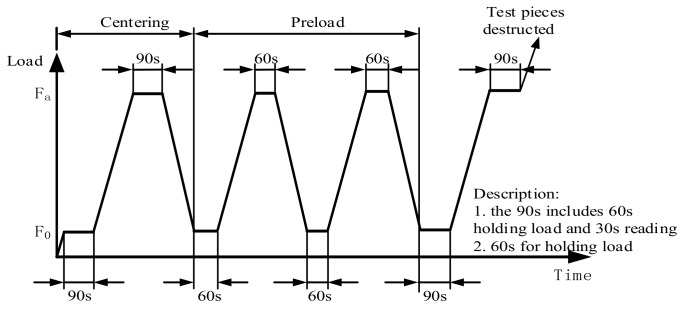
Schematic diagram of elastic modulus test loading.

Subsequently, the load was reduced to the benchmark stress of 0.5 MPa at the same loading speed, and repeated the above process for two times after the loading duration was kept for 60 s. Then the benchmark stress was kept for 60 s, and the deformation at various measurement points was recorded. Subsequently, the load was increased to 1/3 of axial compressive strength at the same speed, the loading duration was kept for 60 s, and the deformation at various measurement points was recorded. Finally, the deformation measuring instrument was removed, and the load was increased at the same speed until the sample was destroyed. The failure load was recorded, and the schematic diagram for loading is shown in Figure 4.

### 2.2. Drying Shrinkage Test

#### 2.2.1. Mixture Ratio

The C40 ordinary concrete was used for the drying shrinkage experiment. The raw materials were the same as the materials for mechanical performance test in the previous section. The mixture ratio of concrete is presented in Table 4. The sand ratio is 35%, and the water-cement ratio (w/t) is 0.49. Four groups numbered as C0, C1, C2 and C3 were made, where C0 was the ordinary concrete without GO, and C0, C1, C2 and C3 were concrete added with 0.02wt%, 0.05wt% and 0.08 wt% GO, respectively. Three samples were included in each group. 

#### 2.2.2. Preparation and Test

**Figure 5:**
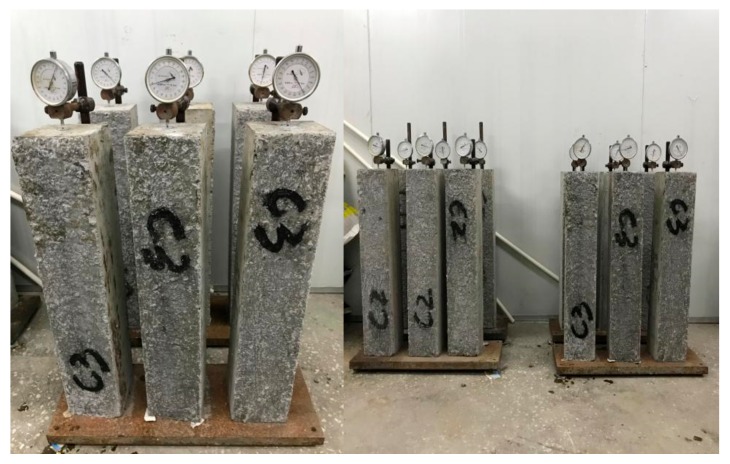
Drying shrinkage test of GO concrete.

The drying shrinkage experiment followed the *Standard for Test Methods of Long-term Performance and Durability of Ordinary Concrete* (GB/T 50082-2009), and the contact method was adopted as the test method. The size of the samples was 100 mm × 100 mm × 515 mm, and the preparation and curing for samples were the same as the conditions for mechanical properties test. The measuring head was affixed to the samples after the mold was removed, and then it was placed in the standard curing room for 3 days. After being taken out, it was immediately put into the drying room under the temperature of (20 ± 2) ℃ and relative humidity of above (60 ± 5) %. The dial indicator was fixed on the standing support, and the support should not be influenced by external vibration. After the dial indicator was installed, the sample was put on the support for reading of the first time, as shown in Figure 5. Shrinkage data was recorded at the ages of 1, 3, 7, 14, 28, 45 and 60 days.

## 3. Experimental Results

### 3.1. Mechanical Properties

#### 3.1.1. Compressive Strength

**Figure 6:**
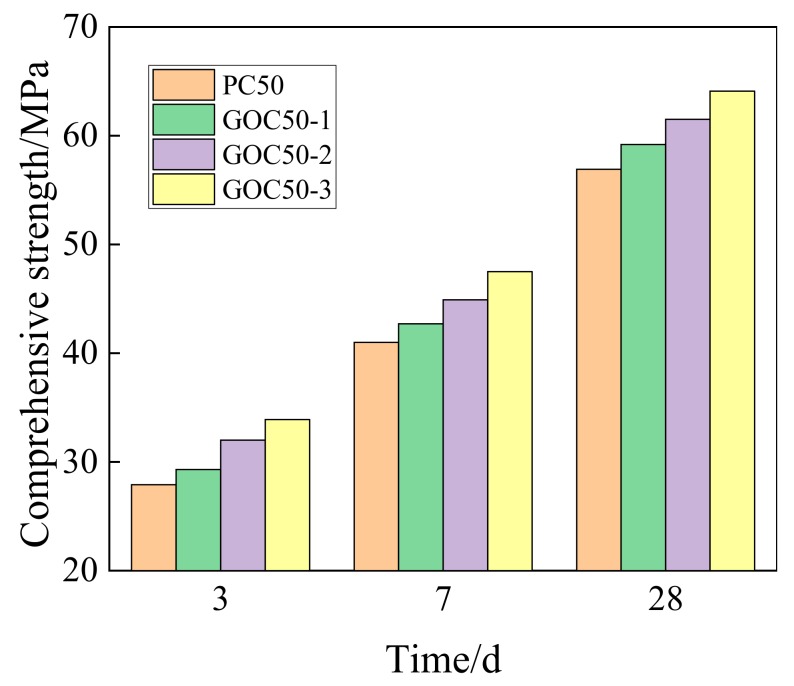
Compressive strength of concrete with different dosage of GO.

Figure 6 presents the change of the compressive strength for concrete with different GO contents at the age of 3, 7, and 28 days. According to the figure, the adding of GO can considerably enhance the compressive strength of concrete. Moreover, the compressive strength of concrete increases with the rise of GO. This trend at different ages (3, 7, and 28 days) is the same. According to the experiment, the compressive strengths of PC50, GOC50-1, GOC50-2 and GOC50-3 samples at the age of 28 days are 56.9 MPa, 59.2 MPa, 61.5 MPa and 64.1 MPa, respectively. 

**Figure 7:**
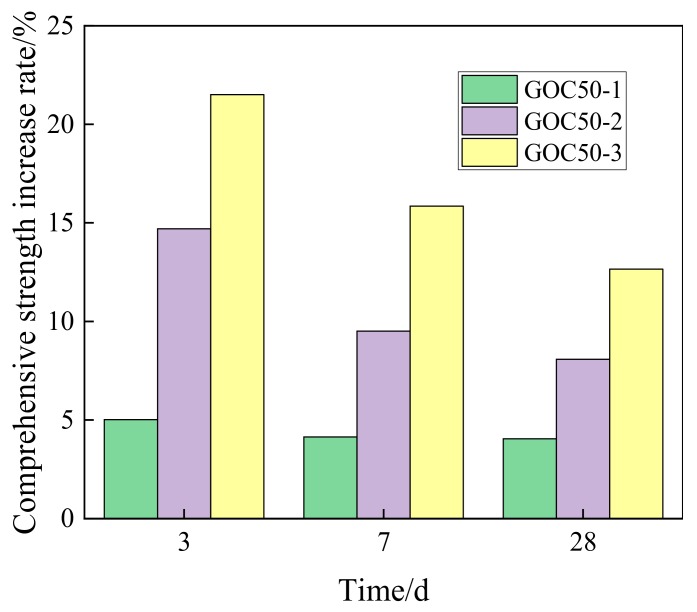
Increase rate of compressive strength for concrete with different dosage of GO.

According to the increasing rate of compressive strength presented in Figure 7, the higher the dosage of GO is, the higher the increasing rate of compressive strength at the same age will be. 

With the increase of age, the influence of GO on the compressive strength of concrete decreases gradually. GOC50-1, GOC50-2 and GOC50-3 samples can increase the compressive strength at the age of 3 days by 5.02%, 14.7% and 21.5% based on PC50, and increase the compressive strength at the age of 28 days by 4.04%, 8.08% and 12.65%, respectively.

#### 3.1.2. Flexural Strength

**Figure 8:**
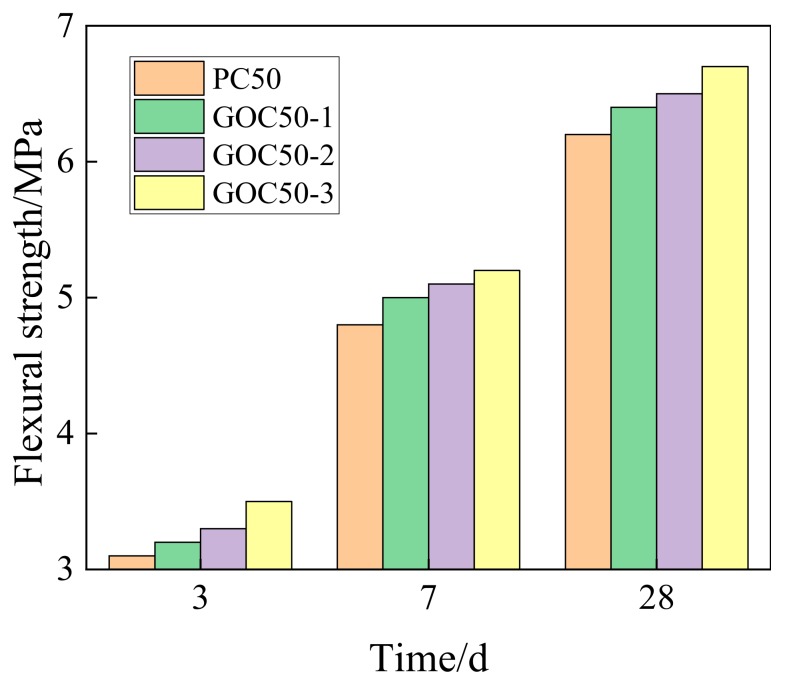
Flexural strength of concrete with different dosage of GO.

Figure 8 shows the comparison of flexural strength at the age of 3 d, 7 d and 28 d under different GO contents. According to the figure, the rule is similar to that of compressive strength. The adding of GO will increase the flexural strength of concrete. Besides, with the rise of the GO dosage, the flexural strength of GO concrete will be further enhanced. According to the test, the flexural strengths of PC50, GOC50-1, GOC50-2 and GOC50-3 samples at the age of 28 days are 6.2 MPa, 6.4 MPa, 6.5 MPa and 6.7 MPa. 

**Figure 9:**
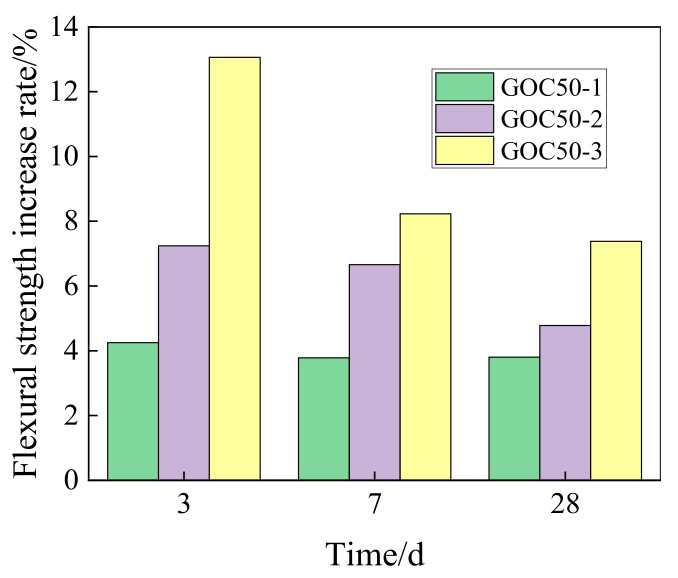
Increase rate of flexural strength for concrete with different dosage of GO.

Figure 9 presents the increasing rate of the flexural strength of GO concrete at various ages when compared with that of concrete without GO. 

GOC50-1, GOC50-2 and GOC50-3 can increase the flexural strength at the age of 3 days by 4.25%, 7.24% and 13.06%, and increase the flexural strength at the age of 28 days by 3.80%, 4.78% and 7.38%. 

Comparatively speaking, the influence of GO on flexural strength is smaller than the influence on compressive strength. For example, at the age of 28 days, GOC50-3 can increase the compressive strength by 12.65% with respect to PC50, but can only increase the flexural strength by 7.38%.

#### 3.1.3. Elasticity Modulus

**Figure 10:**
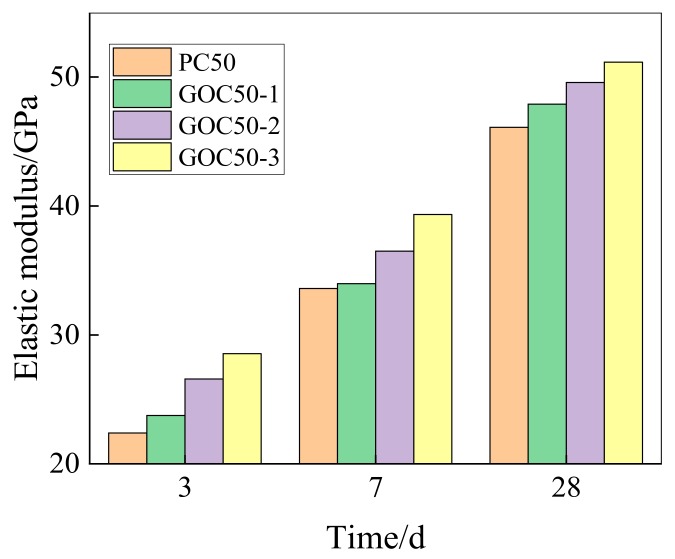
Elastic modulus of concrete with different dosage of GO.

Figure 10 shows the comparison of elasticity modulus between groups of different GO contents and ordinary group at the age of 3, 7 and 28 days. It is observed that the trend is similar to that of compressive strength and flexural strength. The results indicate that GO can considerably enhance the elasticity modulus of concrete. This enhancement is more obviously found for concrete with higher GO. According to the experiment, the elasticity modulus of PC50, GOC50-1, GOC50-2 and GOC50-3 at the age of 28 days is 46.1 GPa, 47.9 GPa, 49.6 GPa and 51.2 GPa. 

**Figure 11:**
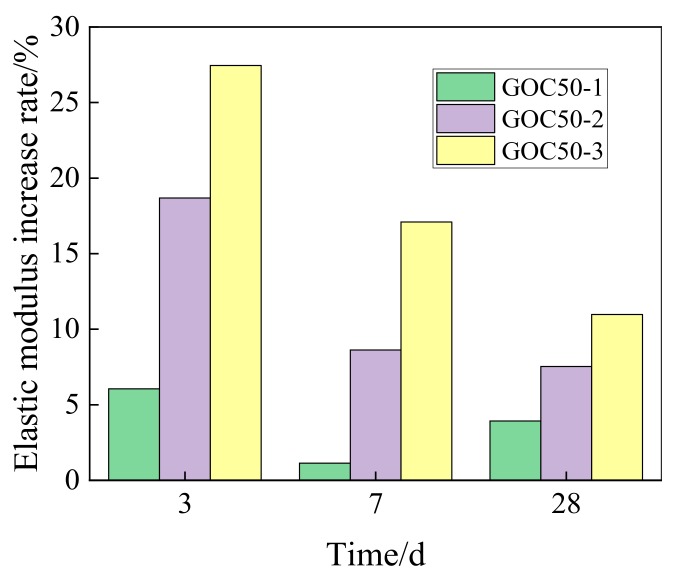
Increase rate of elastic modulus for concrete with different dosage of GO.

Figure 11 shows the increasing rate of the elasticity modulus of GO concrete at various ages when compared with ordinary group. 

According to the figure, the influence of 0.02 wt% GO on the elasticity modulus of concrete decreases first and then increases, and the influence of 0.05 wt% and 0.08 wt% GO on the elasticity modulus of concrete drops with the rise of age.

### 3.2. Drying Shrinkage Effect

**Figure 12:**
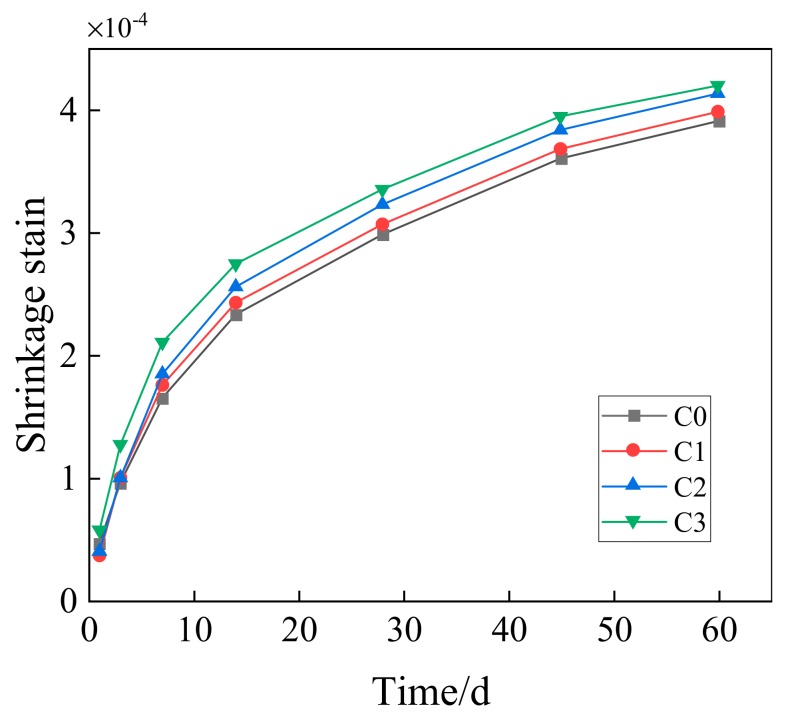
Changes of shrinkage strain with time of GO concrete.

Figure 12 is the changing curve for the shrinking percentage of C0, C1, C2 and C3 concrete. According to the figure, C3 has the largest shrinkage strain during the previous three days. Besides, the shrinkage strains of C1 and C2 are in close agreement with each other, and the shrinkage stain of C0 is higher than that of C1 and C2, but smaller than that of C3. After three days, the shrinkage strain of concrete shows consistent rule that the shrinkage increases with the rise of GO content.

**Figure 13:**
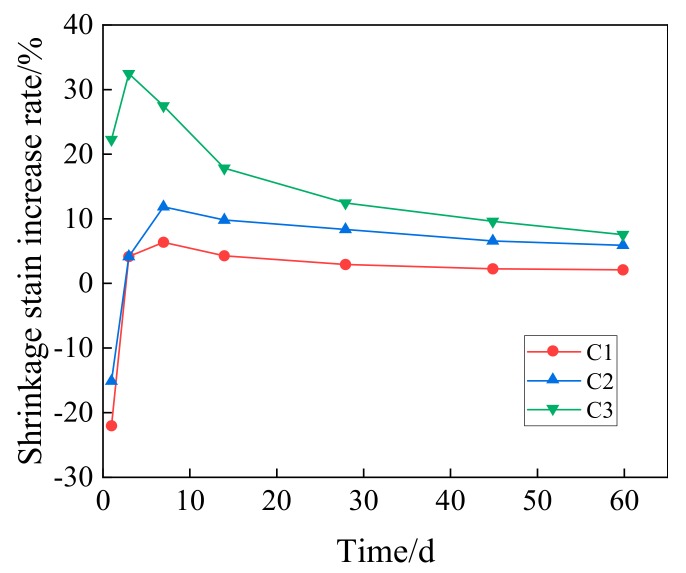
Increase rate with time for the shrinkage strain of GO concrete.

Figure 13 presents the comparison between concrete with different GO contents and ordinary concrete in the increasing rate of shrinkage strain. 

According to the figure, the GO mainly has a big influence on the early-stage shrinkage strain of concrete. On the first day, the shrinkage strain of GO concrete changes within the scope of −22.22–22.22% when compared with that of ordinary concrete. On the third day, the shrinkage strain of concrete with the GO dosage of 0.08 wt% shows the highest increasing rate, which is 32.43%. Later, the shrinkage strain increasing rate of GO concrete in different groups decreases. On the 60th day, the increasing rate of various groups drops to 7.45%. The concrete with the GO dosage of 0.02 wt% has the smallest influence, and the increasing rate of its shrinkage strain with respect to ordinary concrete is only 1.99%.

## 4. Discussion

It is found that the influence of GO nanosheets on the mechanical properties and drying shrinkage of concrete is closely related to the influence of GO on hydration degree, internal microcrack development of concrete and porosity. In the hydration process of concrete, CaO in the cement paste will be hydrated to generate Ca(OH)_2_, which would accelerate hydration by reacting with GO(-COOH) in GO nanosheets [26,27]. When GO nanosheets are just mixed with cement, oxygen functional groups in GO nanosheets which carry negative charges will adsorb metal cations in the cement compound which carry positive charges rapidly to form a flocculent structure. This effect which is called nucleation will reduce the mobility of cement [28]. By measuring the non-evaporable water and calcium hydroxide contents in concrete, Gong et al. [33] have proved that nucleation could enhance the hydration degree of cement. Therefore, GO nanosheets can increase the compressive strength, flexural strength and elasticity modulus of concrete to some extent. Besides, the higher the dosage of GO is, the higher the increasing degree will be.

In the hydration process, more compact crystal structures will be generated in concrete [34], so the early-stage compressive strength, flexural strength and elasticity modulus of concrete will be improved obviously. With the gradual completion of cement hydration reaction, the rate of the interaction will diminish because of the decrease of the contents of the GO(-COOH). So the effect of GO on the strength of composite diminishes with ageing time. The related performance of concrete tends to be stable, and the enhancement effect of GO nanosheets becomes smaller and smaller. Therefore, the increasing rate of compressive strength, flexural strength and elasticity modulus shows a declining tendency. Meanwhile, the influence of 0.02 wt% GO nanosheets on elasticity modulus of concrete decreases first and then increases, which might be related to the increasing rate of its compressive strength.

From the micro perspective, the concrete is divided into aggregate, mortar and interfacial transition zone (ITZ). Some studies have proved that ITZ is the weak phase of mechanical properties of concrete [35]. Therefore, it is speculated that GO nanosheets increases the compressive strength by improving the character of ITZ [21,36], while the improvement of ITZ does not have a huge influence on flexural strength. Moreover, the cohesion brought about by the filling effect of hydrate network structure comprising COO–Ca–OOC and GO nanosheets on calcium silicate hydrate gels can also enhance the compressive strength of concrete [25,37]. As a result, the increasing rate of compressive strength is higher than the increasing rate of flexural strength under the same GO dosage.

The reasons influencing early-stage drying shrinkage of concrete containing GO nanosheets might include the following ones. First, GO nanosheets can increase the hydration degree, reduce the total volume of cement paste, and as a result increase the shrinkage strain. Secondly, Van der Waals force of GO nanosheets under nanoscale will reduce the shrinkage strain [18]. Thirdly, GO nanosheets would hinder the development of microcracks [33], and fill in the microcracks [14]. When the dosage of GO changes, the combined effect produced by the above reasons will also change. Hence, when the age of GO concrete does not exceed 3 days, the shrinking percentage does not present a rule consistent with the dosage.

Existing experiments prove that GO nanosheets could reduce the volume of pores with a diameter of no more than 10 nm, 10-100 nm and above 100 nm in mortar, but increase the number of pores with a diameter of below 10 nm and 10-100 nm [21]. Hence, the composition of pore structure is refined, and the porosity is reduced. Moreover, GO nanosheets can also fill in the gap between hydration products, and further intensify the compaction effect [29]. The older the age is, the more obvious the compaction effect will be. This skeleton structure has increased the rigidity of concrete, and reduced the increasing rate of elasticity modulus and drying shrinkage.

**Figure 14:**
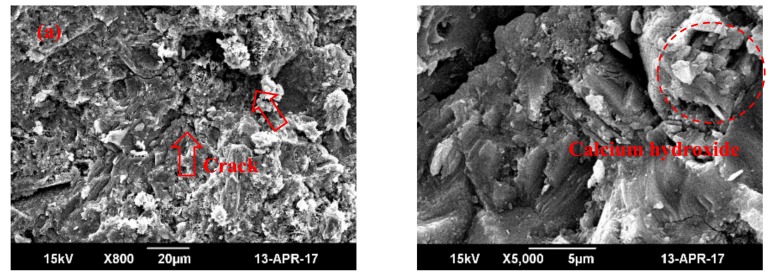

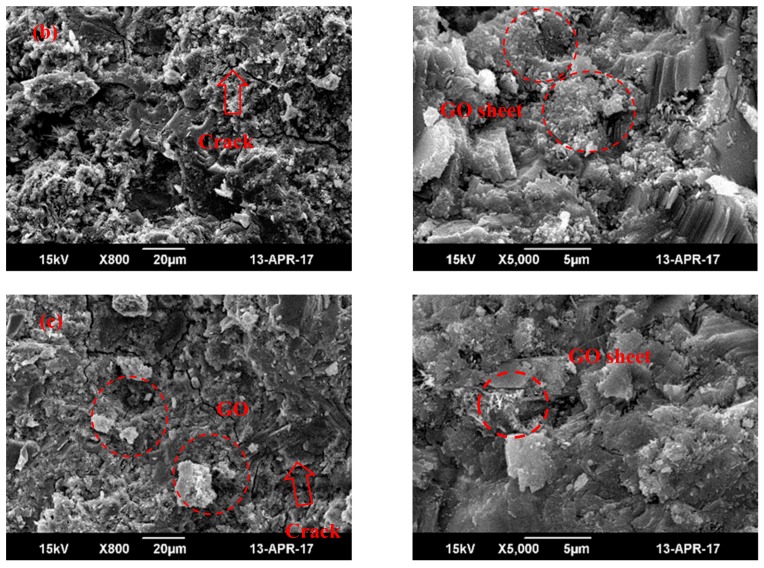
Image of SEM [20]: (**a**) test piece of ordinary cement; (**b**) test piece of GO cement (0.06 wt%); (**c**) test piece of GO cement (1.00 wt%).

In addition, the SEM figure in [20] verifies the above conjecture. According to Figure 14, with the adding of 0.06 wt% GO nanosheets, cracks and pores in the cement paste tend to decrease and shrink, as shown in Figure 14b. In addition, the percentage of GO nanosheets content is relatively low and it is uniformly distributed, so severe gathering, aggregation or granulation signs do not appear. When the dosage of GO nanosheets reaches 1.00 wt%, the number of harmful pores decreases, and the texture structure becomes more compact, as shown in Figure 14c. This shows that the microstructure of cement becomes more compact, the generation of more regular crystalline compounds is promoted, and the cement performance is improved accordingly.

## 5. Concluding Remarks

In this study, the compressive strength, flexural strength and elasticity modulus of concrete containing GO nanosheets, and its dry shrinkage performance have been experimentally investigated. Main findings are summarized as follows:

(1) GO nanosheets can considerably improve the compressive strength at the age of 3, 7 and 28 days. The higher the GO dosage is, the more obvious the strength increase will be. GO nanosheets of different contents can increase the compressive strength of concrete at the age of 28 days by 4.04–12.65%. GO nanosheets can considerably improve the compressive strength of concrete at the age of 3 d the most obviously by 5.02–21.51%.
(2) GO nanosheets can increase the flexural strength of concrete, but the increasing degree is lower than that of compressive strength. Its effect on early-stage strength is comparatively significant, and the most obvious effect happens on the 3rd day (4.25–13.06%). The enhancement effect of flexural strength on the 28th day reaches 3.8–7.38%. Moreover, the flexural strength of concrete will be further improved with the increase of GO nanosheets content.
(3) The enhancement effect of GO nanosheets on early-stage elasticity modulus of concrete is relatively big. The higher the GO content is, the higher the increasing rate of elasticity modulus will be. GO nanosheets can increase the elasticity modulus of concrete at the age of 3 days by 6.05–27.46%, and increase the elasticity modulus at the age of 28 days by 3.92–10.97%.
(4) GO nanosheets will increase the shrinkage strain of ordinary concrete. The higher the content is, the more obvious the enhancement effect will be. GO nanosheets have a comparatively big influence on the increase of early-stage shrinkage strain of ordinary concrete. At the age of 60 days, the GO dosages of 0.02 wt%, 0.05 wt % and 0.08 wt % can increase the shrinkage strain of ordinary concrete by 1.99%, 5.79% and 7.45%, respectively.

## Figures and Tables

**Table 1:** Technical parameters of YAW-2000 compression-testing machine.

Max Test Force (kN)	Relative Error of Test Force Value	Size of Upper and Lower Platens (mm)	Max Gap of Upper and Lower Platens (mm)	Piston Diameter (mm)	Piston Stroke (mm)	Rated Pressure of Hydraulic Pump (MPa)
2000	≤± 1%	3.7 × 3.7	320	250	50	40

**Table 2:** Technical parameters of RE-8060G universal testing machine.

Maximum Load (kN)	Measurement Accuracy	Load Measurement Range	Deformation Measurement Range	Displacement Resolution (mm)	Constant Rate Control of Test Force	Constant Rate Control of Deformation
600	level 1	1–100% FS	1–100% FS	0.002	(1–100%) FS/min	(1–100%) FS/min

**Table 3:** Mixture ratio of GO concrete (kg/m^3^).

Number	Cement	Fly Ash	Silica Fume	Machine-Made Sand	Coarse Aggregate	GO	Water	Polycarboxylate Superplasticizer
PC50	370	70	28	723	1085	0	164	6.08
GOC50-1	370	70	28	723	1085	0.074	164	6.08
GOC50-2	370	70	28	723	1085	0.185	164	6.08
GOC50-3	370	70	28	723	1085	0.296	164	6.08

**Table 4:** Experimental mixture ratio and sample number (kg/m^3^).

Number	Cement	Machine-Made Sand	Coarse Aggregate	GO	Water
C0	398	623	1184	0	195
C1	398	623	1184	0.080	195
C2	398	623	1184	0.199	195
C3	398	623	1184	0.318	195
